# Minithoracotomy Versus Sternotomy for Aortic Valve Replacement: Outcomes from a Latin American Comparative Study

**DOI:** 10.1093/icvts/ivag060

**Published:** 2026-02-24

**Authors:** Josías C Ríos-Ortega, Carlos Quispe-Vizcarra, Josué Sisniegas-Razón, Roger Conde-Moncada, Luisa Talledo-Paredes, Gracia Polo-Lecca, Enrique Velarde-Revilla, Walter Pazos-García, Yemmy Pérez-Valverde, Félix Bocanegra-Silva, Vicente Benites-Zapata, Julio Morón-Castro

**Affiliations:** Cardiovascular Surgery Department, National Cardiovascular Institute, EsSalud, Lima 15072, Peru; Cardiovascular Surgery Department, Edgardo Rebagliati National Hospital, EsSalud, Lima 15072, Peru; Cardiovascular Surgery Department, National Cardiovascular Institute, EsSalud, Lima 15072, Peru; Cardiovascular Surgery Department, National Cardiovascular Institute, EsSalud, Lima 15072, Peru; Cardiovascular Surgery Department, National Cardiovascular Institute, EsSalud, Lima 15072, Peru; Cardiovascular Surgery Department, National Cardiovascular Institute, EsSalud, Lima 15072, Peru; Cardiovascular Surgery Department, Edgardo Rebagliati National Hospital, EsSalud, Lima 15072, Peru; Cardiovascular Surgery Department, Edgardo Rebagliati National Hospital, EsSalud, Lima 15072, Peru; Cardiovascular Surgery Department, National Cardiovascular Institute, EsSalud, Lima 15072, Peru; Cardiovascular Surgery Department, Edgardo Rebagliati National Hospital, EsSalud, Lima 15072, Peru; Unidad de Investigación para la Generación y Síntesis de Evidencias en Salud, Universidad San Ignacio de Loyola, Lima 15026, Peru; Cardiovascular Surgery Department, National Cardiovascular Institute, EsSalud, Lima 15072, Peru

**Keywords:** sortic valve replacement, minimal invasive surgery, Peru

## Abstract

**Objectives:**

We conducted a study comparing full sternotomy (FS) and minithoracotomy (MT) for aortic valve replacement (AVR). The primary end-point was determining all-cause mortality and other variables according to the VARC 3 Consortium.

**Methods:**

Retrospective investigation from January 2017 to December 2024 in 2 referral centres in Peru. We selected 142 patients who were submitted to isolated AVR through MT and 772 through FS. We used unmatched analysis and a propensity score matching (PSM) for matched analysis.

**Results:**

In the unmatched analysis, operative mortality for MT was similar (MT: 2.1% vs FS: 1.6%, *P*: .391), stroke rate in the MT group was 2.1% and in the FS group 1% (*P*: .278), pacemaker insertion was more common in the MT group (MT: 3.5% vs FS: 0.5%, *P* < .001) as well as post-operative atrial fibrillation (POAF) (19% vs 9.2%, *P* < .001). After a PMS, operative mortality was similar (MT: 1/108, 0.9% vs FS: 3/108, 2.8%, *P*: .314); as well as, pacemaker insertion (MT: 2.8% vs FS: 0%, *P*: .081), stroke (MT: 1.9% vs FS: 0%, *P*: .162) or POAF (MT: 15.7%, FS: 8.33%, *P*: .086). At follow-up, PMS analysis showed a similar 5-year survival estimates (MT: 97.6%, IC 95%: 90.7%-99.4% and for FS: 94%, IC 95%: 85.2%-97.6%, *P*: .103).

**Conclusions:**

Isolated AVR through MT or FS has similar operative and follow-up mortality rates. It is possible to implement a minimally invasive cardiac surgery (MICS) program with good results in middle-income countries.

## Graphical Abstract

**Figure:**
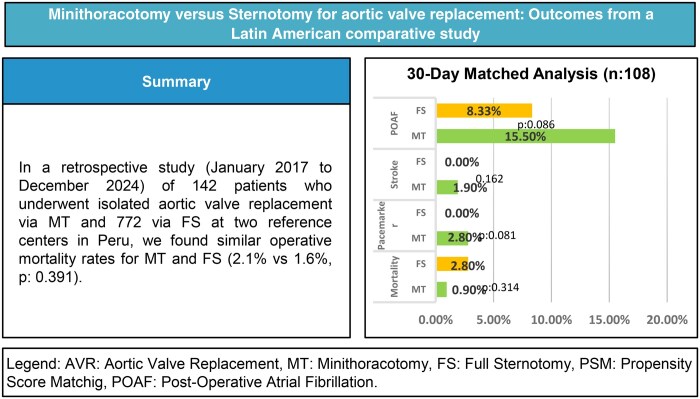

## INTRODUCTION

Aortic valve (AV) disease is the most common valvar heart disease and a major public health problem; in Latin America (LATAM), the prevalence is estimated at 100 cases per 10^3^ and the incidence at 5 cases per 10^3^ inhabitants (year 2019). Surgical aortic valve replacement (AVR) remains the gold standard therapy for severe AV disease in young and low-risk patients.^1^^,^^2^ Full sternotomy (FS) has been the approach of choice; however, FS has grave complications such us disruption, infection (0.3%-5% of cases), and chronic pain.^3^^,^^4^

Surgical approaches have been sought to avoid FS (mini J or T sternotomy, minithoracotomy) since few decades and LATAM was a pioneer in this area.^5^ There are currently many centres around the word that perform minimally invasive cardiac surgery (MICS)—AVR, and this approaches have been shown to reduce pain, hospital stay, post-operative bleeding, and surgical site infectious complications.^6–8^

Latin America has been challenged by health system fragmentation, quality gaps, a growing burden of chronic disease and sociopolitical upheaval.^9^ In this context, developing a MICS program that involves new technologies is a challenge. For example, in our country, to start the MICS program (2010), surgeons had to buy the appropriate instruments; on the other hand, the availability of peripheral cannulas required a long coding process in the social security system.

However, although MICS programs are being developed in some LATAM countries, there are few published studies compared to classic FS. Some single-centre studies compare small series of MT AVR (15-60 patients); or in the other hand, other study show the results of ministernotomy AVR.^10–12^ The importance of our study lies in the fact that it presents the results of AVR through MT (>100 patients) from 2 reference centres in a LATAM country.

## METHODS

### Study population

**Figure 1. Methods for patient selection.**
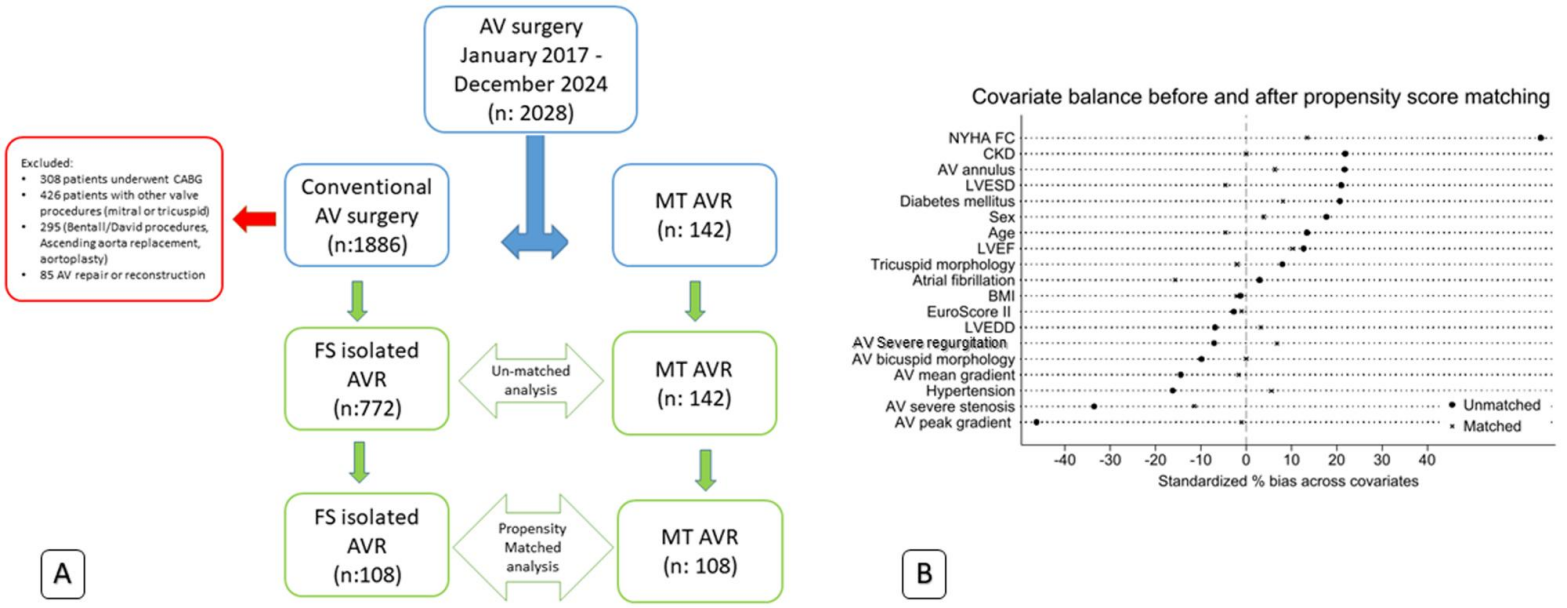
(A) Patients screening diagram. AVR: aortic valve replacement; FS: full sternotomy; MT: minithoracotomy. (B) Covariate balance before and after PSM. NYHA: New York Heart Association; CKD: chronic kidney disease; AV: aortic valve; LVEF: left ventricle ejection fraction; BMI: body mass index; LVEDD: left ventricle end diastolic diameter; LVESS: left ventricle end systolic diameter.

We conducted a retrospective investigation from January 2017 to December 2024 in 2 referral centres (National Cardiovascular Institute and Edgardo Rebagliati National Hospital) in the social security system (EsSalud), Lima, Peru. MT protocol includes patients >18 and <80 years old and excludes morbid obesity, patients underwent to other cardiac procedure, redo, and emergency surgeries. At Rebagliati Hospital, only 1 surgeon performs this procedure, while at National Cardiovascular Institute, 3 surgeons participate. The other surgeons on staff perform the conventional procedure via sternotomy. However, surgeons who perform MICS AVR also perform the same procedure conventionally. **Figure 1A** shows the selection and exclusion of patients.

### Operative technique

All surgeries were performed during cardioplegic arrest on cardiopulmonary bypass (CPB) and underwent intraoperative trans-oesophageal echocardiography (TEE) in addition to standard monitoring for cardiac surgery.

#### Minithoracotomy

We included patients who underwent right MT. We opened the third intercostal space and detached the upper or lower rib from its junction with the sternum. In all cases for CPB, we cannulated right femoral artery and vein. In some cases, video assistance was used. In most cases, automatic knot ties were used (Cor-Knot device). We described our technique in a paper published previously.^13^

#### Full sternotomy

In this approach, we opened the sternum from sternal notch to the xiphoidal appendix, the cannulation strategy was the classic one, ascending aorta for arterial perfusion and atrio-caval for venous drainage.

#### Valve prosthesis

For biological AVR, we use Crown (Livanova) or Hancock (Medtronic) prosthesis; and for mechanical AVR, we use GKS Valve (Beijing Star Medical Devices Co. Ltd.) or CARBOMEDICS (Livanova). The choice of prosthesis depended on the availability provided by the hospital administration.

#### Aortic root enlargement

A heterologous pericardial patch was used. The Nicks Technique was performed in all patient undergoing aortic root enlargement. This procedure was planned according to the calculation of the appropriate prosthesis for the patient’s body surface, with the aim of avoiding the prosthesis/patient mismatch.

### Evaluation stages

We define 3 time periods: (a) Baseline (*t*0): time from hospital admission to before the surgical procedure, (b) perioperative period (*t*1): first 30 post-operative days, and (c) extended follow-up (*t*2): in the unmatched analysis, 33 (IQR: 17-54) months for MT and 25 (IQR: 17-67) months for FS; in the propensity score matching (PSM) analysis, 30 (IQR: 17-54) months for MT and 24 (IQR: 18.5-45.5) months for FS.

Patient follow-up was determined by reviewing the electronic medical records of the Peruvian Social Security (EsSalud) system. In our hospitals, being national referral centres, patients remain for a maximum of 6 months’ post-surgery, after which they return to their original centre; however, through the electronic records, we can follow their progress. According to our protocol, patients must have a transthoracic echo before discharge, a month after surgery, and another before being referred back to their originating centre.

### Outcomes

The primary end-point of the study (observed at *t*1 and *t*2) was all-cause mortality and other variables defined according to the VARC 3 Consortium, which included all strokes (ischaemic or haemorrhagic determined through tomography), any surgical reintervention for structural or non-structural prosthetic valve dysfunction, major bleeding (including types 3 and 4 according to VARC 3), surgical reintervention for endocarditis, permanent pacemaker insertion, post-operative atrial fibrillation (POAF), and perioperative myocardial infarction.^14^

The secondary end-point (observed only at *t*1) included: hospital/ICU stay (calculated from the day of surgery), prolonged mechanical ventilation (>24 hours’ post-surgery), mediastinitis (defined as the need for reoperation due to deep infection), among the echocardiographic findings (determined by transthoracic echocardiography before discharge).

### Statistical analysis

#### Descriptive analysis

We assessed the distribution of continuous variables using complementary graphical and analytical approaches, including inspection of histograms, the Shapiro-Wilk test, comparison of the mean versus the median, and evaluation of skewness and kurtosis. Variables that meet normality criteria are expressed as mean and SD, and those that do not meet them are expressed as median and interquartile range (IQR). Categorical variables are summarized as counts and percentages for baseline, perioperative, and follow-up periods. For mortality and complications, we estimate the cumulative incidence during the perioperative period and throughout follow-up.

#### Comparative analysis

We compared continuous variables that met normality criteria using the Student *t*-test, and those that did not meet normality criteria using the Wilcoxon rank-sum (Mann-Whitney) test. Categorical variables were compared using Pearson’s chi-square test when its assumptions were satisfied; specifically, we verified that no more than 20% of cells had expected counts <5. When this criterion was not met, we used Fisher’s exact test. All hypothesis tests were 2-tailed, with statistical significance set at *P* < .05. No formal multiple-comparison procedures (eg, Bonferroni/Holm) were applied.

#### Propensity score matching

To mitigate baseline confounding, we performed 1:1 PMS. Continuous covariates were modelled using restricted cubic splines with percentile knots at 5, 35, 65, and 95; when percentiles were indistinguishable, knots at 10, 50, and 90 were used. The PSM (probability of receiving the exposure) was estimated via logistic regression including spline terms for continuous covariates (age, BMI, AV annulus diameter, AV peak gradient, aortic valve mean gradient, left ventricular ejection fraction (LVEF), left ventricle end diastolic diameter (LVEDD), left ventricular end systolic diameter (LVESD), and EuroSCORE II) and the following categorical covariates (sex, NYHA functional class III, AV stenosis, bicuspid AV, hypertension, diabetes mellitus, chronic AF, and chronic renal failure), plus a fixed effect for centre. There were no missing data in the covariates included in the PSM model; therefore, no imputation procedures were applied and no observations were excluded due to missingness. Using the logit(PS), we applied nearest-neighbor matching without replacement within a calliper of 0.2 SD of the logit(PS) and restricted analyses to the region of common support. Covariate balance was assessed before and after matching using standardized mean differences (SMDs) and a Love plot (**Figure 1B**); |SMD| < ±10% was considered indicative of adequate balance.

#### Survival analysis

Time-to-event outcomes (eg, mortality) were analysed using 3 complementary Cox proportional hazards approaches. First, we fitted an unadjusted Cox model in the full cohort to estimate crude hazard ratios. Second, as the primary matched analysis, we fitted a Cox model restricted to the propensity score-matched sample, stratified by matched pair with robust standard errors clustered on the matched pair to preserve the matched design. Third, as a sensitivity analysis that retains the full cohort, we fitted a Cox model adjusted for the PSM (included as a continuous covariate). The proportional hazards assumption was assessed using Schoenfeld residuals, both graphically and through formal hypothesis testing, supporting the adequacy of the proportional hazards assumption. Kaplan-Meier (KM) curves were presented by group including 95% CI and numbers at risk, and groups were compared using a stratified log-rank test. In addition, we present a figure with PSM-adjusted survival curves derived from the PS-adjusted Cox model (full cohort) to visually summarize adjusted group differences. All analyses were performed in Stata 18 (StataCorp).

### Ethics

The Institutional Research and Ethics committee approved the protocol of this study (Certificate of Approval 19/2022-CEI). All patients signed informant consent according to health care centre regulation. All data were anonymized. Data were collected and stored solely for the purposes of the present study, with approval from the Ethics Committee of the National Cardiovascular Institute, EsSalud, Lima, Peru.

## RESULTS

### Baseline characteristics

During the study period 142 isolated AVR through MT and 772 through FS were performed (**Table 1**). Between 2020 and 2021, our MICS program was suspended due to the COVID-19 pandemic, hospital resources were diverted to treat Covid patients; cardiac surgeries were only performed via sternotomy (peripheral cannulas and other devices for MICS were not available). The mean age was 64.9 for MT and 66.35 for FS (*P*: .153), respectively. Male sex was most common in the MT group (*P*: .054). AV stenosis was the main indication for surgery and bicuspid valve was the most frequent morphology in both the groups. In the FS group, the median of AV annulus was smaller (22.7 mm, IQR 21-24 vs 23.3 mm, IQR 21-24, *P*: .021). The median of EuroScore II was 0.98% (0.77-1.37) for MT and 1.07% (0.83-1.3) for FS (*P*: .478). **Table 1** also shows the baseline characteristics after the PSM, we can observe the homogenization of the groups.

**Table 1.** Baseline Characteristics Unmatched/PSM Analysis

	Unmatched analysis			PSM analysis		
	MT (*n* = 142)	FS (*n* = 772)	*P*	MT (*n* = 108)	FS (*n* = 108)	*P*
Age (years), mean ± SD	64.9 ± 11.36	66.35 ± 10.38	.153^a^	66.45 ± 10.48	66.95 ± 9.54	.709^a^
Sex, *n* (%)						
Male	96 (68)	454 (59)	**.054** ^b^	71 (65.7)	69 (63.9)	.773^b^
Female	46 (32)	318 (41)		37 (34.3)	39 (36.1)	
BMI (kg/m^2^), mean ± SD	26.54 ± 3.42	26.48 ± 3.22	.861^a^	26.43 ± 3.19	26.5 ± 3.25	.874^a^
Functional class (NYHA), *n* (%)						
II	88 (62)	683 (88)	**.009** ^b^	71 (65.7)	77 (71.3)	.383^b^
III	54 (38)	89 (12)		37 (34.3)	31 (28.7)	
AV disease, *n* (%)						
Stenosis	117 (82)	720 (93)	**.007** ^b^	98 (90.7)	102 (94.4)	.292^b^
Regurgitation	25 (18)	52 (7)		10 (9.3)	6 (5.6)	
AV morphology, *n* (%)						
Bicuspid	94 (66)	545 (71)	.300^b^	74 (68.5)	74 (68.5)	1.000^b^
Tricuspid	48 (34)	227 (29)		34 (31.5)	34 (31.5)	
AV annulus (mm), median (IQR)	23.3 (21-24)	22.7 (21-24)	**.021** ^c^	23.32 ± 2.65	23.2 ± 2.6	.641^a^
≤21 mm, *n* (%)	33 (23)	234 (30)	.082^b^			
AV peak gradient (mmHg), median (IQR)	87.5 (84-110)	96 (70-99)	**.006** ^c^	88.67 ± 30.95	88.9 ± 25.7	.942^a^
AV mean gradient (mmHg), median (IQR)	57 (46-63.5)	55 (41-65)	.700^c^	56.25 ± 20.8	56.6 ± 18	.914^a^
LVEF (%), mean ± SD	60.8 ± 10.07	59.5 ± 10.36	.181^a^	61.25 ± 9.9	60.2 ± 10.43	.452^a^
LVEDD (mm)	43.2 ± 9.12	43.7 ± 5.75	.383^a^	44.75 ± 7.36	44.5 ± 6.74	.793^a^
LVESD (mm)	25.55 ± 8.87	24.03 ± 5.11	**.004** ^a^	26.95 ± 6.77	27.28 ± 6.74	.723^a^
Other basal characteristics, *n* (%)						
Hypertension	61 (43)	395 (51)	.073^b^	44 (40.7)	41 (37.96)	.676^b^
Diabetes	24 (17)	77 (10)	**.021** ^b^	18 (16.7)	15 (13.9)	.559^b^
Chronic atrial fibrillation	5 (4)	24 (3)	.800^b^	5 (4.6)	8 (7.4)	.391^b^
Chronic renal failure	11 (8)	22 (3)	**.004** ^b^	4 (3.7)	4 (3.7)	1.000^b^
EuroScore II (%), median (IQR)	0.98 (0.77-1.37)	1.07 (0.83-1.3)	.478^c^	1.15 ± 0.63	1.16 ± 0.66	.927^b^

a *t*-test.

b Chi-square test.

c Wilcoxon rank-sum (Mann-Whitney) test.

Abbreviations: AV = aortic valve; BMI = body mass index; IQR = interquartile range; kg = kilogram; LVEDD = Left ventricular end diastolic diameter; LVEF = left ventricular ejection fraction; LVESD = Left ventricular end systolic diameter; m = meter; NYHA = New Year Heart Association.

Bold values: statistically significant.

### Surgical procedures characteristics

Cardiopulmonary bypass time (MT: 134 ± 32.2 vs FS: 112 ± 30.4, *P* < .001) and ACC time (MT: 104 ± 25.1 vs FS: 90 ± 24.9, *P* < .001) were longer in the MT group, this difference was also observed after PSM (**Table 2**). Aortic root enlargement was performed in 11% of the MT group and 8% of the FS group. Other surgical characteristics are shown in **Table 2**.

**Table 2.** Surgical Procedures Characteristics

	Unmatched analysis			PSM analysis		
	MT (*n* = 142)	FS (*n* = 772)	*P*	MT (*n* = 108)	FS (*n* = 108)	*P*
CPB time (min), mean ± SD	134 ± 32.2	112 ± 30.4	**<.001** ^a^	133 ± 30.6	115 ± 31.44	**<.001** ^a^
ACC time (min), mean ± SD	104 ± 25.1	90 ± 24.9	**<.001** ^a^	102 ± 24.9	91.38 ± 22.9	**<.001** ^a^
Prosthesis type, *n* (%)						
Mechanical	37 (26)	296 (38)	**.005** ^b^	28 (25.9)	23 (21.3)	.412^b^
Biological	105 (74)	476 (62)		80 (74.1)	85 (78.7)	
Aortic root enlargement, *n* (%)	15 (11)	65 (8)	.400^b^	12 (11.1)	10 (9.3)	.653^b^
Prosthesis size, *n* (%)						
19 mm	16 (11)	81 (10)				
21 mm	51 (36)	281 (36)				
23 mm	50 (35)	304 (39)				
25 mm	23 (16)	99 (13)				
27-29 mm	0 (0)	8 (1.3)				

a *t*-test.

b Chi-square test.

Abbreviations: ACC = aortic cross-clamp; CPB = cardiopulmonary bypass.

Bold values: statistically significant.

### 30 Days evolution

#### Primary end-point

Operative mortality was similar (MT: 2.1%, FS: 1.6%, *P*: .391). One patient in the MT group died of sepsis due to nosocomial pneumonia, and the other 2 suffered intraoperative coronary artery occlusion. This complication was suspected due to the inability to wean the patient from CPB and the evidence of left ventricular motility disorders on TEE. The cause of the occlusion is not described in the medical records, we suspect it was due to a coronary occlusion caused by the prosthesis, since the patients had a bicuspid valve with a small annulus (19 and 21 mm), aortic root enlargement was not performed. This complication occurred in 1 patient per hospital. The procedure was converted to FS, and coronary artery bypass grafting and ECMO support were performed; however, the patients died in the following days due to multiorgan failure. After PSM, mortality was also similar (MT: 0.9% vs FS: 2.8, *P*: .314) (**Table 3**).

**Table 3.** End Points

	Unmatched analysis			PSM analysis		
	MT (*n* = 142)	FS (*n* = 772)	*P*	MT (*n* = 108)	FS (*n* = 108)	*P*
30-days evolution						
Primary end-point^a^						
Total mortality, *n* (%)	3 (2.1)	12 (1.6)	.391^b^	1 (0.9)	3 (2.8)	.314^b^
All stroke (ischaemic/haemorrhagic)	3 (2.1)	8 (1)	.278^b^	2 (1.9)	0 (0)	.162^b^
Redo surgery for structural dysfunction	0 (0)	0 (0)		0 (0)	0 (0)	
Redo surgery for nonstructural dysfunction	0 (0)	2 (0.3)	.543^b^	0 (0)	0 (0)	
Major bleeding (Types 3, 4 according to VARC 3)						
Redo-surgery for excessive bleeding	6 (4.2)	34 (4.4)	.920^b^	3 (2.8)	8 (7.4)	.122^b^
Permanent pacemaker	5 (3.5)	4 (0.5)	**<.001** ^b^	3 (2.8)	0 (0)	.081^b^
POAF	27 (19)	71 (9.2)	**<.001** ^b^	17 (15.7)	9 (8.33)	.086^b^
Perioperative myocardial infarction	1 (0.7)	4 (0.5)	.781^b^	1 (0.9)	0 (0)	.321^b^
Secondary end-point						
ICU stay (days), median (IQR)	3 (2-4)	3 (2-4)	.085^c^	3 (2-4)	3 (2-4)	.312^c^
In hospital stay (days), median (IQR)	10 (8-13)	10 (8-12)	.213^c^	10 (8-13)	9 (7-12)	.332^c^
Other clinical variables, *n* (%)						
Prolonged mechanical ventilation	7 (4.9)	15 (1.9)	.027^b^	6 (5.6)	1 (0.93)	.062^b^
Mediastinitis	0 (0)	0 (0)		0 (0)	0 (0)	
Echocardiographic findings						
Peak AV gradient, median (IQR)	18 (25-32)	18 (24-31)		26 (20-35)	23 (15-31)	
Mean AV gradient, median (IQR)	9 (12-17)	9 (12-16)		14 (10-18)	12 (8-16)	
LVEF, median (IQR)	57 (62-66)	58 (62-66)		60 (57-65)	60 (58-65)	
Follow-up evolution (months), median (IQR)	**33 (17-54)**	**25 (17-67)**	**.632^c^**	**30.5 (17-54)**	**24 (18.5-45.5)**	**.132^c^**
Total mortality, *n* (%)	7 (4.9)	27 (3.5)	.300^b^	2 (1.8)	5 (4.6)	.252^b^
All stroke	4 (2.8)	10 (1.3)	.167^b^	3 (2.8)	1 (0.9)	.312^b^
Redo surgery for structural dysfunction	1 (0.7)	6 (0.7)	1.000^b^	1 (0.9)	0 (0)	.321^b^
Redo surgery for nonstructural dysfunction	1 (0.7)	2 (0.3)	.392^b^	1 (0.9)	0 (0)	.321^b^
Redo surgery for endocarditis	0 (0)	5 (0.6)	.342^b^	0 (0)	1 (0.9)	.321^b^
Permanent pacemaker	5 (3.5)	4 (0.5)	<.001^b^	3 (2.8)	0 (0)	.081^b^

a Definition according to VARC 3.^15^

b Chi-square test.

c Wilcoxon rank-sum test.

Abbreviations: ICU = intensive care unit; LVEF = left ventricular ejection fraction.

Bold values: statistically significant.

Post-operative atrial fibrillation was more frequent in the MT group (19% vs 9.2%, *P* < .001) in the unmatched analysis; after PSM this difference remained but without statistical significance (15.7% vs 8.33%, *P*: .086).

Stroke was similar in both the groups in the unmatched analysis (2.1% vs 1%, *P*: .278) and in the PSM analysis (1.9% vs 0%, *P*: .162).

Permanent pacemaker insertion was more frequent in the MT group (MT: 3.5% vs FS: 0.5%, *P* < .001) in the unmatched analysis, but was similar (2.8% vs 0%, *P*: .081) after PSM.

#### Secondary end points

We found no differences in ICU and hospital stays in both unmatched or matched analysis. Prolonged mechanical ventilation (>24 h) was most frequent in patients who underwent MT (4.9% vs 1.9%, *P*: .027). Redo surgery for bleeding was similar and performed by the same initial approach, no cases of conversion to FS was reported (**Table 3**).

### Follow-up

#### Primary end-point

**Figure 2.**
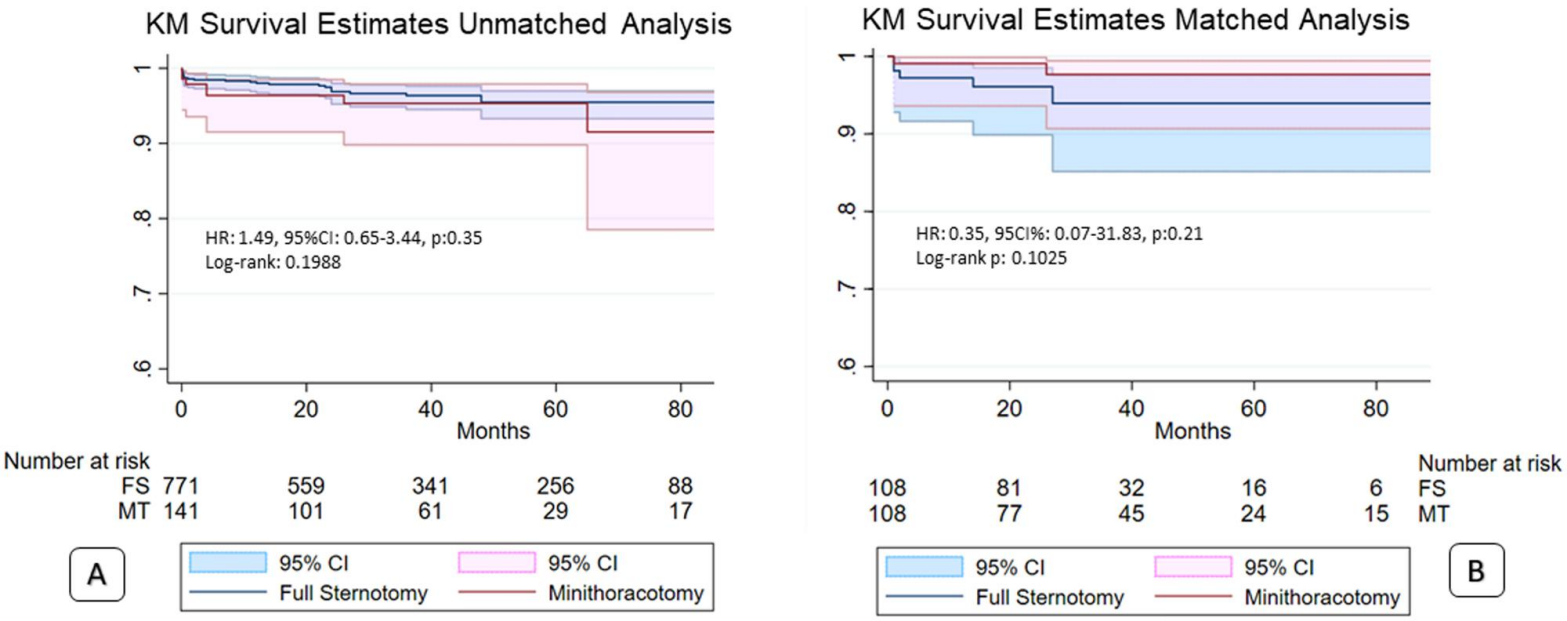
Survival Estimates According to KM Method. (A) Unmatched analysis. (B) Matched analysis. FS: full sternotomy; MT: minithoracotomy

**Figure 3.**
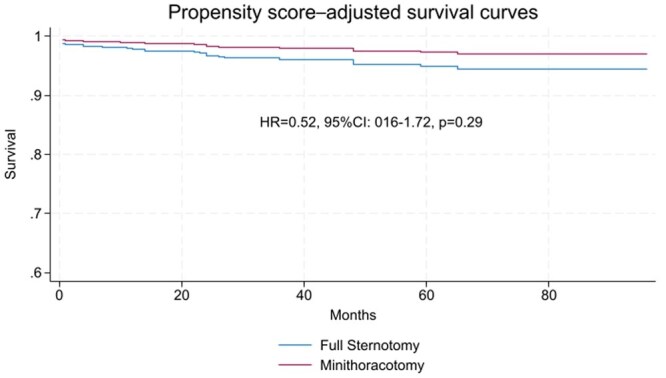
PS-Adjusted Survival Curves According to KM Method

Total mortality was 4.9% for MT and 3.5% for FS (*P* = .300), stroke for MT: 2.8% vs FS: 1.3% (*P*: .167). No other patients required a pacemaker in either group. Other variables are shown in **Table 3**. According to KM method survival estimates were similar (**Figure 2**). In the PMS analysis, 1 year estimated survival for MT: 99.1%, IC 95%: 93.3%-99.9% and for FS: 97.2%, IC 95%: 91.6%-99.1%, *P*: .102. Five-year estimated survival for MT: 97.6%, IC 95%: 90.7%-99.4% and for FS: 94%, IC 95%: 85.2%-97.6%, *P*: .103. As a sensitivity analysis, the propensity score-adjusted Cox model in the full cohort yielded a similar null association, and the corresponding PS-adjusted survival curves (**Figure 3**) showed comparable trajectories, consistent with both the unadjusted full cohort and matched analyses.

## DISCUSSION

### Mortality

A meta-analysis of randomized controlled trials showed that MT has similar operative mortality rates than FS (MT: 2.5% vs FS: 3.1%, RR: 0.83, 95% IC: 0.23-2.83, *P*: .77).^8^^,^^15^ Several studies are published comparing safety and efficacy of the MT with FS, 30-day mortality did not differ significantly and ranged from 0% to 4% for MT and from 1.4% to 5.7% for FS.^15^ A study published in Brazil in 2012 showed an operative mortality for MT AVR of 5% (2/40 patients) and 5% (1/20 patients) for FS.^11^ Our early mortality rates for MT (2.1%) and for FS (1.6%) were similar in both unmatched and matched analysis and acceptable comparing international data. Two of the early deaths in the MT group were due to intraoperative coronary occlusion. This complication can occur acutely after AVR due to inadequate positioning of the valve prosthesis or for acute events such as plaque rupture or embolization.^16^^,^^17^ Proper preoperative planning with multimodal imaging to assess aortic annulus characteristics, coronary artery height, and performing aortic root enlargement in patients with a small annulus could prevent this complication.

### Rhythm disturbances

Permanent pacemaker implantation rate was higher in the MT group in our data. We believe that greater manipulation or traction of the aortic annulus during limited exposure in MT led to this observed difference. However, the pacemaker implantation rate due to rhythm disturbances in MT AVR (unmatched group: 3.5%, matched group: 2.8%) is acceptable and comparable to that reported in previous studies. A meta-analysis of 9 randomized controlled trials found that pacemaker implantation (3.3% vs 4.1%, *P* = .31) was similar in MT vs conventional AVR, other authors found same data.^15^^,^^18^

### Post-operative atrial fibrillation

In several series, MT was associated with 5%-19.9% lower rates of POAF; however, data derived from meta-analysis is controversial. In a systematic review from PSM studies, there was a significantly lower rate of POAF (MT: 11.7% vs FS: 15.9%, *P* = .01); however, in other meta-analysis from randomized controlled trials there was no difference (MT: 13.3%, FS: 8.3%, *P*: .38).^8^^,^^18–20^ In our results, POAF was observed in 19% in the MT group and 9.2% in the FS group (*P* < .001). However, after the PSM analysis, although the difference is not significant (MT: 15.7%, FS: 8.33%, *P*: .089), we believe that this greater tendency to POAF after MT was due to our drain placement protocol in the MT, which we placed inside the pericardial space in direct contact with the right ventricle or right atrium.

### Stroke rate

In our study, stroke rate was 2.1% in the MT group and 1% in the FS group. Regarding this, a meta-analysis reported similar stroke rates in both approaches (2.2% vs 2.2%, RR: 0.99, 95% IC: 0.73-1.34, *P*: .81); however, other studies reported stroke rates from 0.8%-7%.^10^^,^^15^^,^^21^

### Major bleeding

We observed no differences in post-operative bleeding, and redo for bleeding was similar (4.2% vs 4.4%, *P*: .920). Although MT has been associated with a lower rate of bleeding and use of blood components, reoperations due to bleeding are similar in the available data (around 4%-5%), the same as in our case series.^8^^,^^18–21^

### Secondary end points

We no found differences in ICU or hospital stays. It is well established that 1 of the advantages of minimally invasive surgery is rapid recovery and lower hospital stay rates. Some authors reported that patients undergoing MT spent on average 2.1 (1.6-2.7) days less in the hospital, and other authors found that hospital stay was shorter in MT (1.8 [0.92-3.9] days; *P* < .001).^8^^,^^15^^,^^18^ Others series show a median post-operative length of stay for MT: 3-5 days and for FS: 6-8 days.^8^^,^^10^^,^^11^^,^^19^^,^^20^ Our higher hospital stay rate is mainly due to 2 causes: approximately 50% of our patients are from the interior of the country and have nowhere to stay after surgery, and the lack of an early recovery after surgery (ERAS) program. ERAS and fast-track protocols appear viable and have been shown to improve post-operative recovery in MICS. Both ERAS and the fast-track approach allow for a faster and more complete functional recovery, while minimizing perioperative complications.^22^^,^^23^ Regarding this, in our study, prolonged mechanical ventilation was more common in the MT group, in contrast to other authors reported similar rates of respiratory failure (MT: 3.6% vs FS: 5.3%, RR: 0.67, 95% IC: 0.01-2.53, *P*: .181), similar results were published in others papers.^8^^,^^18–21^

### Study limitations

This study has several limitations. First, this is a retrospective study and we included a small number of patients in the MT group, which may suppose a low statistical power and random error. Second, the extended follow-up time was not homogeneous in all participants. Third, medical records were the primary data source, so we cannot guarantee data quality control. Fourth, although a PSM was used, selection bias is significant (prosthesis type, surgeon experience, etc). Fifth, the number of events for several outcomes, particularly within the matched cohort, was limited, reducing statistical power and yielding imprecise estimates with wide confidence intervals. This limitation is partly inherent to 1:1 PSM, which discards patients without suitable matches and further decreases the effective sample size and event counts. Accordingly, non-significant findings should not be interpreted as evidence of equivalence or no effect, and the possibility of Type II error cannot be excluded. Although the propensity score-adjusted Cox sensitivity analysis using the full cohort showed results consistent with the unadjusted and matched analyses, residual confounding due to unmeasured factors and model specification cannot be fully ruled out. Overall, results should be interpreted cautiously and confirmed in larger studies with more events.

On the other hand, our study has several strengths, it is 1 of the few to show comparative data from a middle-income country, and our study shows that it is possible, even with limited resources, to implement programs to innovate in cardiac surgery.

### Future perspectives

Our AVR results through MT are acceptable; however, it can be improved by incorporating an ERAS protocol, which can help us reduce ICU and hospital stays. We have implemented a post-surgical drain placement protocol that avoids contact with cardiac structures, it is placed only in the pleural space, leaving the pericardium partially open for blood drainage through the pleura. We expect this to reduce the frequency of POAF.

## CONCLUSION

In a LATAM experience, both approaches, MT and FS for AVR, have similar and acceptable mortality and stroke rates. MT has a greater propensity for POAF or need for a pacemaker.

## Data Availability

Authors declare the availability of data.

## ABBREVIATIONS

AVR: aortic valve replacement
FS: full median sternotomy
ICU: intensive care unit
IQR: interquartile range
KM: Kaplan-Meier
MICS: minimally invasive cardiac surgery
LATAM: Latin American
MT: minithoracotomy
NYHA: New York Heart Association
POAF: post-operative atrial fibrillation
PSM: propensity score matching
